# Association between specific antiarrhythmic drug prescription in the post-procedural blanking period and recurrent atrial arrhythmias after catheter ablation for atrial fibrillation

**DOI:** 10.1371/journal.pone.0253266

**Published:** 2021-06-24

**Authors:** Chaitanya L. Malladi, Douglas Darden, Omar Aldaas, Praneet S. Mylavarapu, Michael Eskander, Florentino Lupercio, Frederick T. Han, Kurt S. Hoffmayer, Farshad Raissi, Gordon Ho, David Krummen, Gregory K. Feld, Jonathan C. Hsu

**Affiliations:** Division of Cardiology, Department of Medicine, University of California, San Diego, La Jolla, CA, United States of America; Ohio State University, UNITED STATES

## Abstract

**Purpose:**

To evaluate if specific AADs prescribed in the blanking period (BP) after catheter ablation of atrial fibrillation (AF) may be associated with reduced risk of early recurrence (ER) and/or late recurrence (LR) of atrial arrhythmias.

**Methods:**

A total of 478 patients undergoing first-time ablation at a single institution were included. Outcomes were: ER, LR, discontinuation of AAD less than 90 days post-ablation, and second ablation. ER was defined as AF, atrial flutter (AFL), or atrial tachycardia (AT) > 30 seconds within BP. LR was defined as AF/AFL/AT > 30 seconds after BP.

**Results:**

Of 478 patients, 14.9% were prescribed no AAD, 26.4% propafenone/flecainide, 34.5% sotalol/dofetilide, 10.7% dronedarone, and 13.6% amiodarone. Patients prescribed amiodarone were more likely to have persistent AF, hypertension, diabetes, and other comorbidities. In unadjusted analyses, there were no differences between groups in relation to ER (log rank P = 0.171), discontinuation of AAD before ninety days post-ablation (log rank P = 0.235), or freedom from second ablation (log rank P = 0.147). After multivariable adjustment, patients prescribed amiodarone or dronedarone were more likely to experience LR than those prescribed no AAD [Adjusted Hazard Ratio (AHR) 1.83, 95% CI 1.10–3.04, p = 0.02; AHR 1.79, 95% CI 1.05–3.05, p = 0.03, respectively].

**Conclusion:**

Following first-time catheter ablation, there were no differences between specific AAD prescription and risk of ER, while those prescribed amiodarone or dronedarone in the BP were more likely to experience LR than those prescribed no AAD, which may represent an association due to confounding by indication.

## Introduction

Catheter ablation has been established as an effective treatment for drug-refractory symptomatic AF [1]. However, patients undergoing ablation often experience early recurrence (ER) of atrial tachyarrhythmias within the first three months after the procedure [1]. ER is not considered ablation failure, as many patients who experience ER will not develop late recurrence (LR) [2]. ER may result from transient post-procedure inflammation, autonomic imbalance, or incomplete formation of durable lesions [3]. Therefore, consensus guidelines have established a 3-month blanking period (BP) post-ablation in which recurrence of atrial tachyarrhythmias should not prompt reintervention [1].

Multiple studies have shown that ER after first-time pulmonary vein isolation (PVI) ablation is strongly predictive of LR [4–6]. Consequently, clinicians frequently use AAD in the BP in order to maintain sinus rhythm, control symptoms, and allow remodeling. The specific AAD drugs are often chosen empirically with limited data supporting a particular AAD over others. Several randomized controlled trials have evaluated the effects of AAD post-ablation compared to no AAD [7–12]. The 5A, EAST-AF, and AMIO-CAT trials all found that AAD reduced ER, but did not affect LR. However, these trials did not compare the efficacies of different classes of AADs, and the longest period of follow-up between all these studies was one year. Meta-analyses have also confirmed the limited efficacy of AADs at maintaining sinus rhythm beyond the blanking period [13–15]. A retrospective analysis of patients in a large national claims database evaluated efficacy of different AADs by examining readmission rate following ablation, but only followed patients for ninety days after their procedures [16]. Thus, overall, these studies do not provide guidance on whether to use an AAD, or which specific AAD agent to use and are limited in long-term follow-up.

In this study, we have categorized and evaluated the association between type of AAD used in the BP after first-time PVI ablation and the following outcomes: ER of atrial tachyarrhythmias, LR of atrial tachyarrhythmias, time to initial discontinuation of AAD, and freedom from second ablation. Our study will also aim to clarify if the anticipated reduction in ER associated with AAD use in the BP based on RCT data correlates with reductions in LR of atrial arrhythmias.

## Methods

### Study design and registry population

This is an observational single-center cohort study based on data from the University of California, San Diego (UCSD) AF Ablation Registry, which has been approved by the UCSD Institutional Review Board. The UCSD AF Ablation Registry was designed as a clinical registry of all patients undergoing left atrial ablation procedures at UCSD. We obtained IRB approval to collate data from the electronic medical record (EMR), thereby waiving the need for individual consent. Patient, provider, and intra-procedural data was collected through a procedural database (Perminova Inc, San Diego, CA). All AF Ablation procedures captured by the registry from October 2009 to March 2015 (n = 847) were linked to clinical encounters recorded at UCSD Medical Center through the EMR system (Epic, Verona, WI). Patients with a prior AF ablation procedure (n = 300) or less than three months of follow-up clinical encounters (n = 69) recorded in the UCSD EMR were excluded.

### Patient characteristics

Data regarding baseline demographics, medical history, laboratory data, and medications were collected as a part of the UCSD AF Ablation registry. The following covariates were based on the most recent provider documentation prior to ablation: age, sex, body mass index (BMI), and type of AF (paroxysmal vs. persistent). Other elements of the medical history [hypertension (HTN), hyperlipidemia (HLD), end-stage renal disease (ESRD), diabetes mellitus (DM), obstructive sleep apnea (OSA), prior cerebrovascular accident (CVA)/transient ischemic attack (TIA), chronic obstructive pulmonary disease (COPD), congestive heart failure (CHF), coronary artery disease (CAD), smoking] were collected from reviewing patients’ charts up to the date of ablation.

### Radiofrequency ablation procedure

Informed consent was obtained prior to all ablation procedures. General anesthesia was used in all cases. Intravenous heparin was administered as a bolus followed by a continuous infusion with a goal activated clotting time of 300–400 seconds. Transseptal puncture was performed under direct visualization with intracardiac echocardiography. Electroanatomic mapping systems were used in all cases (CARTO (Biosense-Webster Inc, Diamond Bar, CA) or Ensite, St Jude Medical, Inc, Minneapolis, MN). Esophageal position and temperature were monitored during all left atrial ablations using a multipolar temperature probe (Circa S-Cath, Englewood, CO) at the level of the ablation catheter to avoid any temperature rise above 38°C. Following initial ablation using a segmental, circumferential or combined approaches, the patient was observed for a period of 30 minutes after the last ablation, for evidence of pulmonary vein (PV) conduction recovery. If recovery of PV conduction was observed, repeat PV isolation was performed using similar approaches. Additional lesion sets outside the PVs were performed at the discretion of the operator, including left atrial roof line, mitral valve isthmus line, coronary sinus ablation, and complex fractional atrial electrogram ablations. Additionally, organized atrial arrhythmias that occurred after initial ablation, such as AFL or AT, were mapped and ablated, as were ectopic premature atrial contractions. Closed and open-irrigated, and non-contact and contact force sensing catheters were also used at the discretion of the operator. The endpoint of PVI ablation was elimination of all PV potentials and demonstration of entrance and exit block by pacing after a 30-minute waiting period, elimination of all premature atrial contractions that might trigger AF, and demonstration of bidirectional conduction block across any linear ablations, if adjunctive ablations were performed. It was not standard practice to induce AF before or after ablation at our institution. Adenosine was not used in all patients, so, this was not a part of our formal protocol. Success of pulmonary vein isolation was confirmed by demonstration of bidirectional block.

After ablation, patients were hospitalized overnight and usually discharged the day after the procedure. Oral anticoagulation was restarted in all patients following ablation for a minimum of 2 months and continued thereafter at the discretion of the treating physician primarily depending on the patient’s CHADS2-VASc score.

### Patient groups and outcomes

Patients were grouped based on specific prescribed AAD drugs (or no AAD) immediately after ablation. Five groups were used in accordance with a previous retrospective study [16]: 1) no AAD; 2) propafenone/flecainide; 3) sotalol/dofetilide; 4) dronedarone; 5) amiodarone.

Outcomes included: freedom from AF/atrial flutter (AFL)/atrial tachycardia (AT) within the 3-month blanking period (ER), freedom from AF/AFL/AT after the 3-month blanking period (LR), time to discontinuation of AAD, and freedom from second ablation. Recurrence of AF/AFL/AT was defined as AF, AFL, or AT lasting > 30 seconds on 12-lead ECG, ambulatory monitoring, or implantable device, as recommended by contemporary guidelines [1]. As a part of the registry, follow-up arrhythmia monitoring was pre-specified, and a 12-lead ECG was obtained at each follow-up visit. Routine follow-up ambulatory ECG monitoring (i.e. 7–14 day event monitor) was attempted in all patients at 6 months, 1 year, and 2 years after ablation. Moreover, in the presence of suggestive symptoms, additional ambulatory ECG monitoring was recommended, which was consistent with consensus guidelines at the time of the registry. Based on results from ECGs, event and Holter monitors, recurrences of atrial arrhythmias were documented in the medical record and tracked for our registry. Time to discontinuation of AAD was calculated as the time from the ablation procedure to initial discontinuation of antiarrhythmic therapy. Lastly, whether the patient underwent a second ablation procedure was determined based on the EMR at UCSD. Repeat ablations documented in the chart but not performed at UCSD were also included in this outcome variable.

### Statistical analysis

Categorical variables were reported as count and percentage. Continuous variables were shown as means ± one standard deviation for normally distributed variables and as medians with 25^th^ and 75^th^ percentiles for non-normally distributed variables. Categorical variables across multiple groups were compared using chi-square or Fisher’s exact test (if expected cell counts were less than five). Continuous variables across multiple categories were analyzed by analysis of variance (normally-distributed) or the Kruskal-Wallis test (non-normally distributed).

All four outcomes (ER of atrial arrhythmias, LR of atrial arrhythmias, time to discontinuation of AAD, and freedom from second ablation) were analyzed using the Kaplan-Meier method, and a log-rank value of significance was calculated. Patients who were lost to follow-up were censored at the date of last known follow-up. A 3-month blanking period was used for analyzing the recurrence of atrial arrhythmias and time to discontinuation of AAD. Unadjusted and adjusted Cox proportional hazards ratios were computed and presented as hazard ratios (HR) with 95% confidence intervals (CI). Variables for adjustment were decided *a priori* and included the following as potential confounders: age, sex, BMI, paroxysmal/persistent AF, HTN, HLD, ESRD, DM, OSA, prior CVA/TIA, COPD, CHF, CHADS2-VASc, CAD, smoking. In particular, age was an important variable included in our multivariate analyses, as the recurrence and incidence of AF increases significantly with age [17]. Missing values were minimal and roughly equivalent between groups for all variables and were therefore omitted. Analyses were performed using Stata 11 (StataCorp, LLC, College Station, TX) statistical software. All p-values were analyzed as two-tailed. A p-value less than 0.05 was considered statistically significant.

## Results

### Patient characteristics

A total of 478 patients were analyzed in this study with baseline characteristics summarized in Table 1. The median follow-up times for the five groups (no AAD, propafenone/flecainide, sotalol/dofetilide, dronedarone, amiodarone) were 37.5 months, 40.0 months, 42.3 months, 34.7 months, and 52.2 months, respectively. There was no statistically significant difference in median follow-up (p = 0.269) between groups. The median durations of AAD therapy for the four groups excluding no AAD (propafenone/flecainide, sotalol/dofetilide, dronedarone, amiodarone) were 90 days, 108 days, 107 days and 126 days, respectively. There was no statistically significant difference in median duration of AAD therapy (p = 0.200) between groups.

**Table 1:** Baseline patient characteristics stratified by choice of AAD immediately after PVI ablation for AF.

		No AAD (n = 71)	Propafenone /Flecainide (n = 126)	Sotalol /Dofetilide (n = 165)	Dronedarone (n = 51)	Amiodarone (n = 65)	p-value
**Age [mean (SD)]**		62.6 ± 11.1	61.2 ± 9.0	65.3 ± 9.5	65.8 ± 9.3	67.5 ± 8.7	<0.001
**Sex (male)**		48 (68%)	75 (60%)	122 (74%)	35 (69%)	41 (63%)	0.120
**Body mass index [median (IQR)]**		27.8 (25.0–30.0)	27.3 (23.9–30.3)	28.4 (25.8–32.3)	28.3 (25.1–33.1)	29.2 (26.4–34.8)	0.004
**Type of AF**							<0.001
	**Paroxysmal**	51 (72%)	113 (90%)	96 (58%)	35 (69%)	30 (46%)	
	**Persistent**	20 (28%)	13 (10%)	69 (42%)	16 (31%)	35 (54%)	
**Hypertension**		34 (47%)	59 (47%)	101 (61%)	30 (59%)	47 (73%)	0.003
**Hyperlipidemia**		30 (42%)	49 (39%)	69 (42%)	15 (29%)	36 (56%)	0.057
**End-stage renal disease**		2 (3%)	0 (0%)	0 (0%)	0 (0%)	0 (0%)	0.051
**Diabetes mellitus**		6 (8%)	3 (2%)	22 (13%)	5 (10%)	14 (22%)	0.001
**Obstructive sleep apnea**		5 (7%)	12 (10%)	26 (16%)	4 (8%)	10 (15%)	0.181
**Prior cerebrovascular accident/transient ischemic attack**		5 (7%)	12 (10%)	13 (8%)	3 (6%)	7 (11%)	0.856
**Chronic obstructive pulmonary disease**		2 (3%)	3 (2%)	7 (4%)	0 (0%)	4 (6%)	0.422
**Congestive heart failure**		8 (11%)	1 (1%)	13 (8%)	2 (4%)	11 (17%)	<0.001
**CHADS2-VASc2 [median (IQR)]**		2 (1–3)	1 (1–2)	2 (1–3)	2 (0–3)	3 (1–4)	<0.001
**Coronary artery disease**		12 (17%)	6 (5%)	31 (19%)	5 (10%)	24 (37%)	<0.001
**Smoking**		12 (17%)	15 (12%)	35 (21%)	7 (14%)	21 (33%)	0.009

There were significant differences in baseline patient characteristics (Table 1). Patients prescribed amiodarone were older (p < 0.001), had higher median BMI (p = 0.004), were more likely to be smokers (p = 0.009), and were more likely to have hypertension (p = 0.003), diabetes (p = 0.001), CHF (p < 0.001), and higher CHADS2-VASc2 scores (p < 0.001). Patients prescribed propafenone or flecainide were more likely to have paroxysmal AF (p < 0.001) and less likely to have either CAD (p < 0.001) or CHF (p < 0.001).

In S1 Table, we report the AAD prescriptions in our cohort before and after ablation. Patients prescribed an AAD pre-ablation were frequently continued on the same AAD post-procedure, while those prescribed no AAD pre-ablation were often prescribed an AAD post-procedure.

### Ablation outcomes

**Fig 1:**
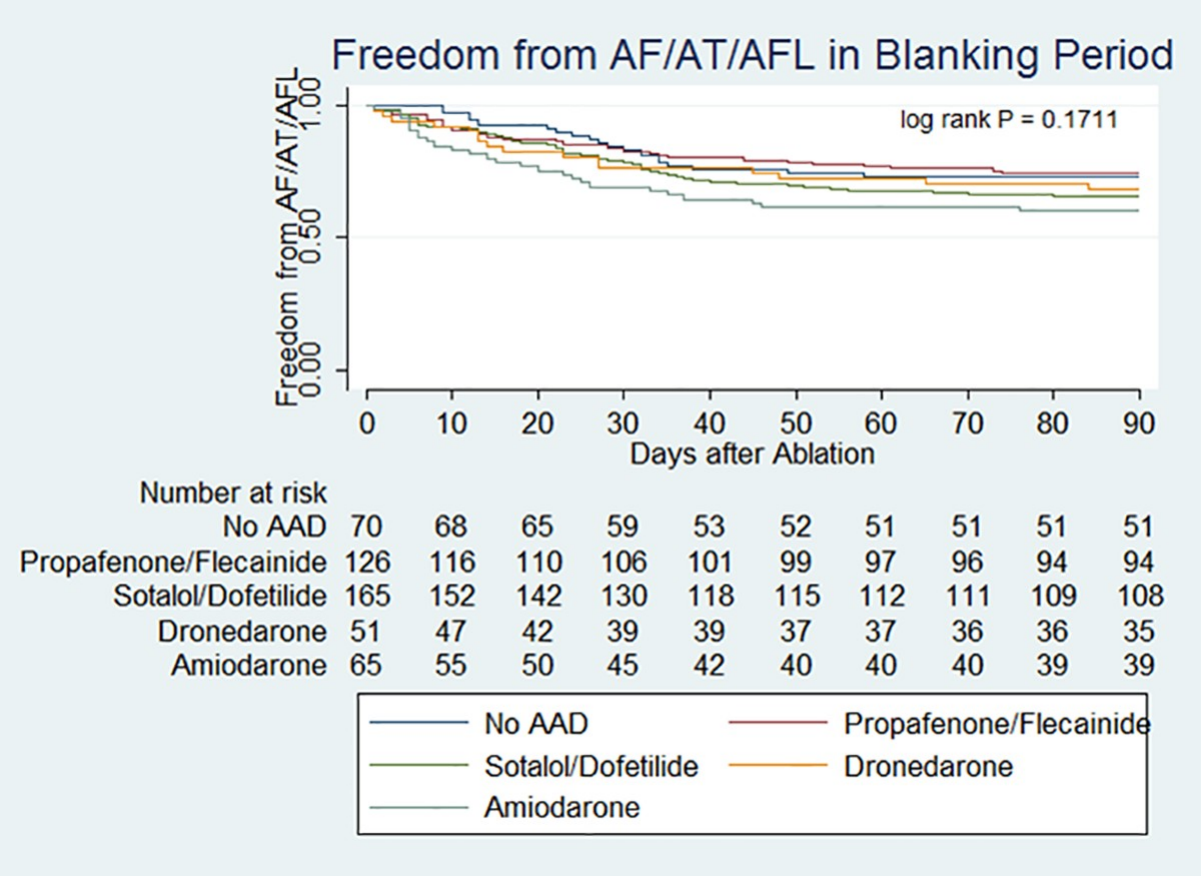
Kaplan-Meier plot of freedom from AF/AT/AFL in 3-month blanking period after ablation.

Kaplan-Meier (KM) survival curves analyzing time to early recurrence of AF/AFL/AT between groups is shown in Fig 1. In univariate analysis, there were no significant differences between AAD groups in the risk of early recurrence of AF/AFL/AT (log rank P = 0.1711). Compared to those patients who were prescribed no AAD, there was no difference in the risk of ER among different groups of AADs in unadjusted and adjusted multivariable analyses (Table 2). A total of 10/71 (14%) of patients who were not on AAD immediately after ablation were prescribed an AAD within the 3-month blanking period. Of those prescribed an AAD during the blanking period, four were prescribed flecainide, four were prescribed sotalol, one was prescribed dofetilide and one amiodarone.

**Table 2:** Unadjusted and adjusted Cox proportional hazard ratios for early recurrence: AF/AFL/AT recurrence within three-month blanking period (No AAD is reference group).

	Unadjusted HR + 95% CI	p-value	Adjusted HR + 95% CI	p-value
**No antiarrhythmic drug**	(Reference)	--	(Reference)	--
**Propafenone/Flecainide**	0.95 (0.54–1.68)	0.859	1.06 (0.58–1.94)	0.847
**Sotalol/Dofetilide**	1.35 (0.80–2.27)	0.255	1.11 (0.64–1.92)	0.715
**Dronedarone**	1.23 (0.63–2.38)	0.548	1.08 (0.54–2.17)	0.833
**Amiodarone**	1.71 (0.95–3.09)	0.076	1.42 (0.74–2.73)	0.286

**Fig 2:**
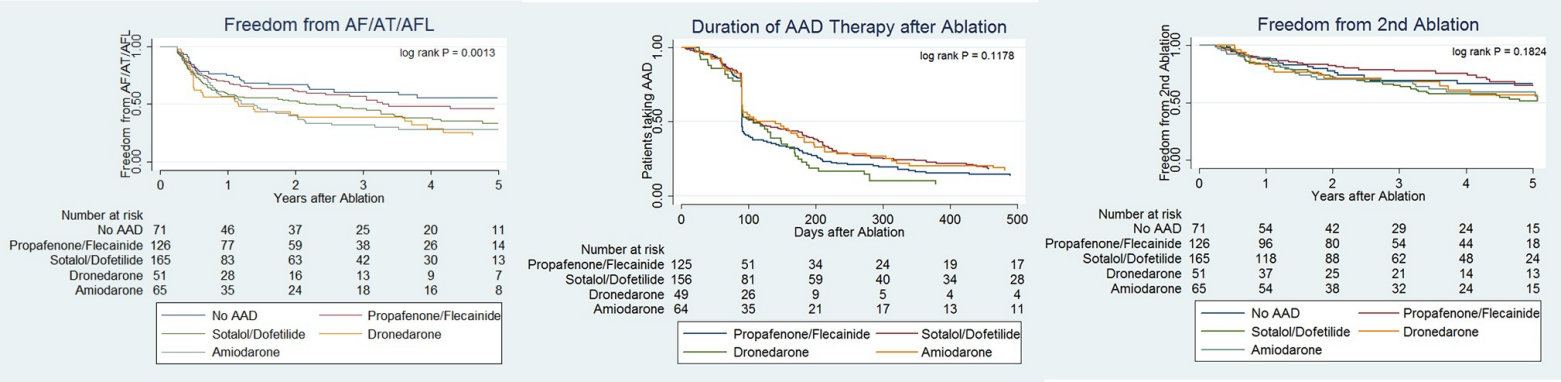
Kaplan-Meier plots of A) recurrence of atrial arrhythmias (excluding a 3-month post-procedural blanking period) on or off AAD; B) time to initial discontinuation of AAD; C) time to second ablation procedure (excluding a 3-month post-procedural blanking period).

For evaluating LR of AF/AFL/AT, the group of patients prescribed no AAD had the strongest association with avoiding recurrence of atrial arrhythmias, while patients prescribed amiodarone were the most likely to experience recurrence (Fig 2A; log rank P = 0.001). Patients prescribed dronedarone or amiodarone were more likely to experience recurrent AF/AFL/AT compared to those not on AAD post-ablation in both unadjusted and adjusted multivariable analyses (Table 3). After adjustment, patients prescribed dronedarone had an AHR of 1.79 (95% CI 1.05–3.05; p = 0.032) and patients prescribed amiodarone had an AHR of 1.83 (95% CI 1.10–3.04; p = 0.020) compared to those not prescribed AAD. The comparison between patients prescribed sotalol/dofetilide compared to those prescribed no AAD did not reach statistical significance after adjustment [AHR 1.52 (0.97–2.38); p = 0.070]. Those prescribed propafenone/flecainide had the least increase in risk of recurrence compared to no AAD [HR 1.15 (0.74–1.81); AHR 1.16 (0.72–1.88)]. However, these associations did not reach statistical significance in both unadjusted (p = 0.531) and adjusted analyses (p = 0.550).

**Table 3:** Unadjusted and adjusted Cox proportional hazard ratios for late recurrence: AF/AFL/AT recurrence excluding three-month blanking period (No AAD is reference group).

	Unadjusted HR + 95% CI	p-value	Adjusted HR + 95% CI	p-value
**No antiarrhythmic drug**	(Reference)	--	(Reference)	--
**Propafenone/Flecainide**	1.15 (0.74–1.81)	0.531	1.16 (0.72–1.88)	0.550
**Sotalol/Dofetilide**	1.69 (1.11–2.56)	0.014	1.52 (0.97–2.38)	0.070
**Dronedarone**	2.01 (1.23–3.27)	0.005	1.79 (1.05–3.05)	0.032
**Amiodarone**	2.09 (1.32–3.30)	0.002	1.83 (1.10–3.04)	0.020

With regards to duration of AAD therapy after ablation, all groups had a significant proportion of their AAD prescriptions discontinued approximately three months after the procedure. Although the patients prescribed propafenone and flecainide were less likely to be continued on AAD past the 3-month blanking period post-ablation, this association did not reach statistical significance when comparing the outcome amongst all groups (Fig 2B; log rank P = 0.235). In unadjusted and adjusted multivariable analyses (Table 4), none of the AAD groups were more or less likely to be discontinued by 90 days in comparison to amiodarone: propafenone/flecainide AHR 1.17 (0.68–1.99), p = 0.571; sotalol/dofetilide AHR 1.02 (0.63–1.65), p = 0.927; dronedarone AHR 1.14 (0.62–2.08), p = 0.671.

**Table 4:** Unadjusted and adjusted Cox proportional hazard ratios for: Discontinuation of AAD before 90 days after ablation (Amiodarone is reference group).

	Unadjusted HR + 95% CI	p-value	Adjusted HR + 95% CI	p-value
**Amiodarone**	(Reference)	--	(Reference)	--
**Propafenone/Flecainide**	1.42 (0.91–2.21)	0.120	1.17 (0.68–1.99)	0.571
**Sotalol/Dofetilide**	1.07 (0.69–1.67)	0.763	1.02 (0.63–1.65)	0.927
**Dronedarone**	1.17 (0.67–2.05)	0.570	1.14 (0.62–2.08)	0.671

Lastly, with regards to time to second ablation, in unadjusted and adjusted multivariable analyses, we found no difference in freedom from second ablation amongst the various AAD groups in individual comparisons to the group prescribed no AAD in the BP (Table 5).

**Table 5:** Unadjusted and adjusted Cox proportional hazard ratios and 95% confidence intervals for: Second ablation procedure (No AAD is reference group).

	Unadjusted HR + 95% CI	p-value	Adjusted HR + 95% CI	p-value
**No antiarrhythmic drug**	(Reference)	--	(Reference)	--
**Propafenone/Flecainide**	0.95 (0.54–1.68)	0.860	0.81 (0.45–1.46)	0.482
**Sotalol/Dofetilide**	1.52 (0.91–2.55)	0.112	1.33 (0.76–2.32)	0.318
**Dronedarone**	1.34 (0.71–2.53)	0.374	1.08 (0.54–2.15)	0.825
**Amiodarone**	1.54 (0.86–2.76)	0.146	1.41 (0.74–2.71)	0.300

## Discussion

In this single center, retrospective study, we found that patients prescribed amiodarone or dronedarone during the blanking period were more likely to experience LR of atrial arrhythmias than those prescribed no AAD during the blanking period. This association is likely the result of patient selection in these AAD groups and confounding by indication of prescription of these medications. Firstly, the amiodarone group had the highest percentage of persistent AF patients. These patients are more likely to experience recurrence of atrial arrhythmias after ablation [18, 19]. Secondly, provider choice of selecting amiodarone as an AAD drug could be driven by the presence of other significant comorbidities interfering with choosing other drugs (significant CAD, CHF, age, QT interval). Guidelines provide a Class I recommendation that amiodarone only be considered after other agents have failed or have significant contraindications for a particular patient [20]. However, in our analyses, the association between amiodarone use in the blanking period and LR maintained statistical significance even after adjustment for patient comorbidities, age, and classification of AF (paroxysmal versus persistent), suggesting that there may be unmeasured variables confounding this association.

Patients prescribed dronedarone did not have higher rates of these comorbidities in our study, yet they were more likely to experience LR post-ablation compared to those prescribed no AAD. Prospective analyses and larger cohorts may be required to confirm a relationship between choice of dronedarone during the blanking period and increased risk of recurrence. However, it is also possible that this finding results from confounding by indication for dronedarone prescription. Regardless, the associations between dronedarone and amiodarone demonstrated here merit further consideration for whether prescription of these AADs in the BP has a deleterious effect in the long-term. Darkner et al. found no significant difference in LR for amiodarone compared to placebo at 6 months of follow-up post-ablation [9]. However, the duration of follow-up for the data presented in our cohort is considerably longer, as the median follow-up in the amiodarone group was 52 months.

With regards to ER, we found that none of the AAD groups were more likely to experience ER than those prescribed no AAD during the BP. This finding does not necessarily contradict those of the aforementioned randomized clinical trials [7–10], as this is a retrospective cohort. Rather, the lack of an association between any of the studied AAD groups and reduced rates of ER in our study could suggest equivalent benefit from any AAD (or possibly no AAD) post-ablation.

Additionally, we found there was no significant difference between choice of AAD during the 3-month blanking period post ablation and duration of post-ablation AAD therapy. While it did not reach statistical significance, there was a trend towards earlier discontinuation of AAD in the dronedarone and propafenone/flecainide groups compared to the amiodarone and sotalol/dofetilide groups. Overall, however, the data presented here are inconsistent with concerns that particular AADs may be tougher to wean than others, controlling for patient comorbidities and other risk factors for recurrence of AF. Similarly, with regards to requiring a second ablation, we did not find a significant difference between AAD groups in Kaplan-Meier, unadjusted, or adjusted multivariable analyses suggesting that no specific AAD appears superior in avoiding subsequent need for ablation.

As a whole, these results do not unequivocally suggest an association between choice of AAD in the blanking period and LR. Rather, underlying patient comorbidities and triggers for recurrence could be responsible for LR after first-time ablation. However, the association demonstrated here between amiodarone or dronedarone and increased risk of LR suggests that patients who are prescribed these medications may be more likely to have other comorbidities or confounding attributes that are also associated with LR. This finding also highlights the need for future investigations in pathophysiological mechanisms of recurrence of atrial tachyarrhythmias more than one year post-ablation, and whether choice of AAD therapy alters the natural progression of recurrence of AF after ablation.

### Study limitations

The current analysis has several inherent limitations. Due to the non-randomized, retrospective design of our study, we cannot rule out bias being introduced due to residual confounding, despite extensive adjustment for potential confounders. Patients prescribed no AAD in the BP had fewer comorbidities associated with AF recurrence, especially compared to patients prescribed amiodarone or dronedarone. These findings suggest that confounding by indication may likely explain the associations with recurrent AF in follow-up. Moreover, the association seen between AAD therapy and recurrent AF may be a result of effect-cause instead of cause-effect. However, given the long duration of follow-up in our study, these findings also suggest that optimization of comorbidities may play an important role in preventing late recurrence of atrial arrhythmias. Additionally, we highlight the need for future prospective analyses, evaluating the initiation of specific AAD and its effect on recurrent AF in the early and late time periods.

Secondly, the outcome for evaluating discontinuation of AAD may not accurately represent the most important outcome for patients, as patients may be restarted on AAD shortly after discontinuation if recurrent AF is found. Lastly, there are relative contraindications and indications for particular AADs that will primarily drive provider choice (i.e. Class 1C in unstable CAD, or dronedarone in heart failure) [20]. This effect is corroborated by our findings that patients prescribed propafenone/flecainide were less likely to have CAD or CHF at baseline prior to ablation (Table 1).

However, understanding relative risk of recurrence between appropriate choices of AAD can still provide valuable information to providers. Furthermore, randomized studies comparing individual choices between groups (i.e. propafenone vs. flecainide, or dofetilide vs. sotalol) can provide even more evidence for the efficacy of particular choices within Vaughn-Williams classes.

## Conclusion

Based on this registry analysis, among the different AAD groups studied, we found no difference with regards to ER of atrial tachyarrhythmias, time to initial discontinuation of AAD, and requirement of second ablation. Patients prescribed amiodarone or dronedarone in the blanking period were more likely to experience late recurrence of atrial tachyarrhythmias compared to those on no AAD, which may represent an association based on confounding by indication.

## Supporting information

S1 Table AAD prescriptions pre-ablation versus AAD prescriptions post-ablation. (DOCX)

S1 File (DO)

S2 File (DO)

S3 File (DO)

S4 File (DO)

S5 File (DO)

S6 File (DO)

S7 File (DO)

S8 File (DO)

S9 File (DO)

S10 File (DO)
