# Supplementary material for: Association between specific antiarrhythmic drug prescription in the post-procedural blanking period and recurrent atrial arrhythmias after catheter ablation for atrial fibrillation
Source: PLoS One. 2021 Jun 24;16(6):e0253266. doi: 10.1371/journal.pone.0253266 (PMC8224843; doi:10.1371/journal.pone.0253266)
Supplement: S1 Table — (DOCX) [file pone.0253266.s001.docx]

**S1 Table.** AAD prescriptions pre-ablation versus AAD prescriptions post-ablation.

|  | **Post-ablation** | | | | | |  |
| --- | --- | --- | --- | --- | --- | --- | --- |
| **Pre-ablation** |  | Amiodarone | Propafenone/  Flecainide | Sotalol/  Dofetilide | Dronedarone | No AAD | Total |
|  | Amiodarone | 34 | 3 | 5 | 0 | 9 | 51 |
|  | Propafenone/  Flecainide | 6 | 78 | 21 | 4 | 9 | 118 |
|  | Sotalol/  Dofetilide | 8 | 11 | 86 | 3 | 6 | 114 |
|  | Dronedarone | 2 | 1 | 6 | 36 | 1 | 46 |
|  | No AAD | 16 | 33 | 50 | 9 | 44 | 155 |
|  | Total | 66 | 126 | 168 | 52 | 69 |  |
